# Current and new rotavirus vaccines

**DOI:** 10.1097/QCO.0000000000000572

**Published:** 2019-09-05

**Authors:** Rachel M. Burke, Jacqueline E. Tate, Carl D. Kirkwood, A. Duncan Steele, Umesh D. Parashar

**Affiliations:** a Viral Gastroenteritis Branch, Centers for Disease Control and Prevention, Atlanta, Georgia; b Enteric and Diarrheal Diseases, Global Health, Bill and Melinda Gates Foundation, Seattle, Washington, USA

**Keywords:** immunization, pediatric gastroenteritis, rotavirus, rotavirus vaccines

## Abstract

As of 2019, four rotavirus vaccines have been prequalified by the WHO for use worldwide. This review highlights current knowledge regarding rotavirus vaccines available, and provides a brief summary of the rotavirus vaccine pipeline.

**Recent findings:**

Data generated from use of currently available products supports their effectiveness and impact in diverse settings. Rotavirus vaccines have a favorable risk–benefit profile, but previous associations of rotavirus vaccination with intussusception necessitate continued monitoring for this rare but serious adverse event. Implementation of rotavirus vaccines was jeopardized in late 2018 and 2019 by a shortage of vaccine supply. Fortunately, with the prequalification of two additional vaccines in 2018, countries have increased choice in products with different characteristics, pricing, and implementation strategies. Other vaccines currently in development may open up further immunization strategies, such as neonatal vaccination schedules or parenteral administration.

**Summary:**

Rotavirus vaccines have demonstrated impact in reducing diarrheal morbidity and mortality worldwide. As countries begin to introduce the newly prequalified vaccines, additional data will become available on the safety and effectiveness of those products. Products in the pipeline have distinct profiles and could be an essential part of the expansion of rotavirus vaccine use worldwide.

## INTRODUCTION

Rotavirus is one of the leading causes of severe pediatric diarrhea globally, and is associated with ∼125 000–200 000 deaths each year in children under 5 years [1^▪▪^,^2^,^3^]; approximately half of all rotavirus-associated deaths occur in just four countries (India, Nigeria, Pakistan, and the Democratic Republic of the Congo) [3]. The WHO recommends rotavirus vaccination as part of an integrated package of prevention-oriented and treatment-oriented interventions to reduce diarrheal morbidity and mortality [4]. In 2006, two rotavirus vaccines – Rotarix (GlaxoSmithKline Biologicals SA, Rixensart, Belgium) and RotaTeq (Merck & Co., Inc., West Point, PA, USA) – were licensed and almost immediately introduced into the national immunization programs of several countries. In 2009, after the live-attenuated, oral vaccines had been shown to be efficacious in developing countries in Africa and Asia, WHO recommended rotavirus vaccines for priority inclusion in national immunization programs worldwide [4]. Currently, rotavirus vaccines are in wide use globally, and have made a demonstrable impact on the burden of disease [5]. Nonetheless, opportunities for growth in global coverage remain. The prequalification of two products previously available only on a national level may help to alleviate global rotavirus vaccine supply constraints [6^▪^,^7^]. This review provides a brief summary of the current status of rotavirus vaccine introductions globally, available knowledge on the impact and safety of rotavirus vaccines, and future directions in rotavirus vaccine development. 

**Box 1:**
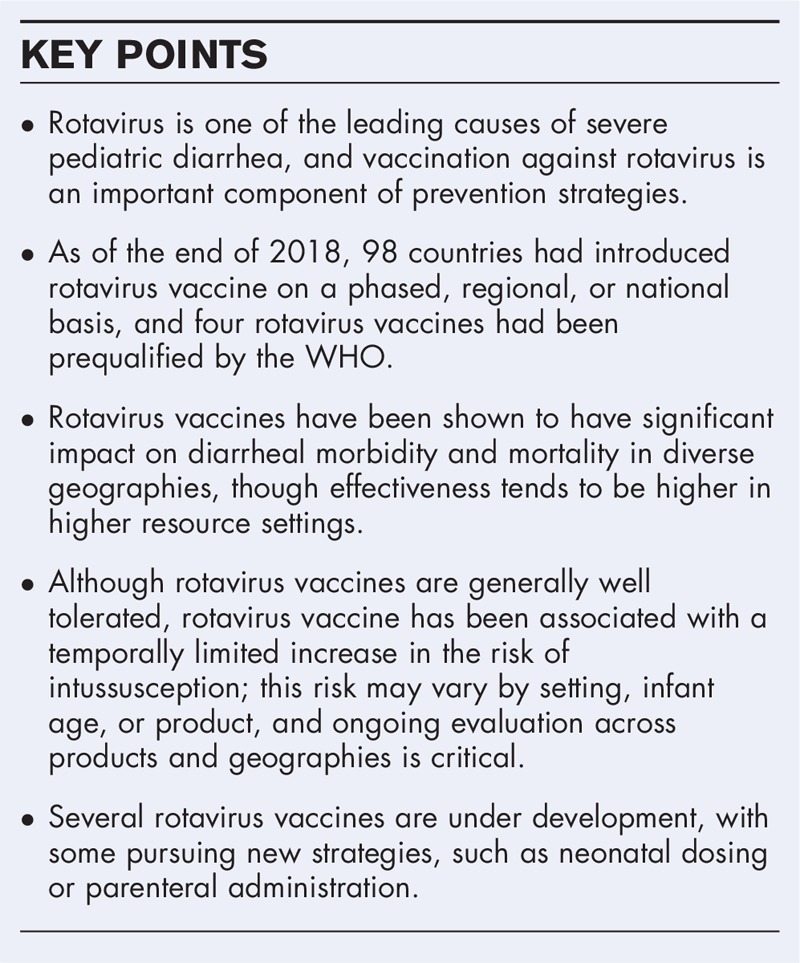
no caption available

## CURRENT STATUS OF ROTAVIRUS VACCINE INTRODUCTIONS GLOBALLY

**FIGURE 1:**
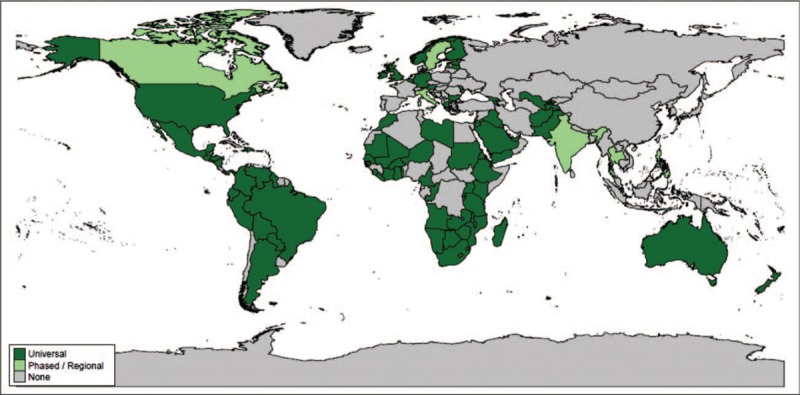
Map of rotavirus vaccine introduction worldwide. The status of rotavirus vaccine introduction is indicated by color, with dark green for universal (national) introduction, and light green for introduction on a phased or regional basis. Countries that have not introduced rotavirus vaccine into their national immunization schedules are shown in gray; rotavirus vaccine may be available via the private market in some countries.

By the end of 2018, 92 countries worldwide had introduced rotavirus vaccine into their national immunization programs, and an additional 6 countries had introduced rotavirus vaccine on a phased or regional basis [8] (Fig. 1). In many lower income countries, rotavirus vaccine introduction is supported by Gavi, The Vaccine Alliance. Of the original 73 Gavi-eligible countries, 46 have received Gavi support for rotavirus vaccine introduction as of 2019, whereas 8 had been currently Gavi-approved and were planning for introduction in the near future [8].

Four rotavirus vaccines are prequalified by WHO: Rotarix (GlaxoSmithKline Biologicals; prequalified in 2009), RotaTeq (Merck & Co., Inc.; prequalified in 2008), Rotavac (Bharat Biotech, Hyderabad, India; prequalified in 2018), and ROTASIIL (Serum Institute of India PVT. LTD., Pune, India; prequalified in 2018) [9]. However, the most recently prequalified vaccines -- Rotavac and ROTASIIL -- are currently only in use in India (both vaccines) and Palestine (Rotavac only). Elsewhere, as of the end of 2018, 74 countries were using Rotarix in their national immunization programs, 14 were using RotaTeq, and 9 were using both Rotarix and RotaTeq [8].

Two additional rotavirus vaccines are available on a national basis: Rotavin-M1 [Center for Research and Production of Vaccines and Biologicals (POLYVAC), Hanoi, Vietnam], available on the private market in Vietnam, and the Lanzhou Lamb Rotavirus (LLR) vaccine (Lanzhou Institute of Biological Products Co., Ltd., Lanzhou, China), available on the private market in China [10]. Each vaccine has a unique presentation and profile, resulting in distinct considerations for introduction (Table 1).

**Table 1:** Characteristics of currently licensed, live, oral rotavirus vaccines

Product	Manufacturer	WHO PQ?	Doses	Composition	Formulation/storage	Presentation
Globally licensed						
Rotarix	GSK	Y	3	G1P[8]	Liquid 2–8 °C for 36 months	One-dose plastic tube | Strip of 5-single-dose plastic tubes
RotaTeq	Merck	Y	2	G1, G2, G3, G4, P[8]	Liquid 2–8 °C for 24 months	One-dose plastic tube
Rotavac	Bharat Biologicals	Y	3	G9P[11]	Liquid frozen −20 °C long-term | 2–8 °C for 7 months^a^	5-dose or 10-dose glass vial, with dose dropper
ROTASIIL	Serum Institute of India	Y	3	G1, G2, G3, G4, G9	Lyophilized < 25 °C for 30 months | <40 °C for 18 months^b^	One-dose or two-dose glass vial, with vial of diluent, adapter, and syringe
Nationally licensed						
Rotavin-M1	POLYVAC	N	3	G1P[8]	Liquid frozen -20 °C for 24 months | 2–8 °C for 2 months	One-dose vial
Lanzhou Lamb Rotavirus Vaccine	Lanzhou Institute of Biological Products	N	1 annually age 2 months to 3 years	G10P[15]	Liquid	Vial

^a^Nonfrozen presentation expected in 2020 but not yet prequalified.

^b^Liquid presentation expected in 2020 but not yet prequalified.

## ROTAVIRUS VACCINES IN GLOBAL USE AS OF MID-2019: ROTARIX AND ROTATEQ

### Impact and effectiveness

In 2019, we will mark 10 years since WHO first recommended rotavirus vaccination for children worldwide [4]. Since that time, data on the impact and effectiveness of rotavirus vaccines have continued to be generated in diverse settings. These data are especially important for countries transitioning from Gavi support to self-financing, as they provide evidence-based rationale for continued support of rotavirus vaccination.

The Americas, on the basis of existing efficacy data for the region and consequent WHO recommendation, began to introduce rotavirus vaccines soon after licensure in 2006 [11^▪^]. A 2018 meta-analysis of studies from Latin America estimated a vaccine effectiveness of 71% (95% confidence interval (CI) 61–79%) against rotavirus hospitalization in children less than 12 months of age in the region [11^▪^]. This same review calculated a median reduction of 43% [interquartile range (IQR) 37–50%] in acute gastroenteritis death rates in children less than 1 year in low-mortality countries, and 45% (IQR: 30–55%) in high-mortality countries. The authors further estimated that in 2015, rotavirus vaccination averted a median of ∼123 000 rotavirus-associated hospitalizations and ∼660 rotavirus-associated deaths in the 15 Latin American countries that have introduced rotavirus vaccine, and ∼2260 rotavirus-associated hospitalizations and ∼180 rotavirus-associated deaths in the two rotavirus vaccine-using Caribbean countries. A review and meta-analysis of rotavirus vaccine impact and effectiveness in the United States found a median vaccine effectiveness of 84% (IQR: 83–91%) against rotavirus-associated hospitalizations or emergency department visits [12^▪^]. Vaccine effectiveness estimated via meta-analysis (using mixed effects models) were similar for RV5 (84%) and RV1 (83%).

Although rotavirus vaccines are available on the private market in many countries in the WHO European region, only 18 of 53 have introduced rotavirus into their national vaccine program [8]. Vaccine effectiveness estimates for the region vary, with the highest effectiveness seen in higher resource countries [e.g. 86% (95% CI: 83–89%) against rotavirus hospitalization in German children less than 5 years [13], and 94% (95% CI: 80–98%) against rotavirus hospitalization in Finnish children eligible for vaccination [14]), and lower effectiveness seen in lower resource countries [e.g. 62% (95% CI: 36–77%) against rotavirus hospitalization in Armenian children less than 2 years [15], and 79% [95% CI: 62–88%] against rotavirus hospitalization in children less than 2 years in the Republic of Moldova [16]].

In the WHO African region, 35 of 47 countries have introduced rotavirus vaccine into their national immunization schedules as of 2018, and several more are planning for imminent introduction [8]. Of seven African countries in the WHO Eastern Mediterranean region (EMRO), four have introduced the vaccine [17]. In 2005, the African Rotavirus Surveillance Network was established to document the rotavirus disease burden in Africa; by 2013, there were 22 countries participating in the network [18^▪^]. Data from this network were recently analyzed to compare rotavirus burden in countries that had introduced the vaccine before 2013, versus those countries who had yet to introduce rotavirus vaccine by 2015 [18^▪^]. Rotavirus positivity declined significantly over time in countries that were early introducers of the vaccine (35% in 2010 to 19% in 2015 for countries introducing before 2013); the decline was less marked for countries introducing after 2013 (44% in 2010 to 25% in 2015). Countries that had not introduced the vaccine by the end of the study period showed no significant change in rotavirus positivity (32% in 2010 and 30% in 2015) [18^▪^]. Other recently published data from the region showed estimates of vaccine effectiveness ranging from 49 to 86%, with the greatest effectiveness observed against severe disease and in younger infants [19–22].

In Asia, only eight countries have introduced rotavirus vaccine on a national basis; two additional countries (India and Pakistan) have begun a phased introduction [23^▪^]. A recent review and analysis of data from this region found a median vaccine effectiveness of 94% in low child mortality countries, 64% in medium child mortality countries, and 49% in high child mortality countries [23^▪^]. This analysis further estimated that universal introduction of rotavirus vaccine in all 43 countries in the studied region could avert 710 000 rotavirus hospitalizations and 35 000 rotavirus deaths annually, assuming an achieved coverage equal to that of the third dose of diphtheria–tetanus–pertussis (DTaP) vaccines [23^▪^]. Other recently published studies from this region found vaccine effectiveness in similar ranges by child mortality stratum: 80% in Japan [24] (low child mortality), 60% in the Philippines [25] (high child mortality).

### Safety

In randomized controlled trials, rotavirus vaccines have been well tolerated [26–31]; a Cochrane review of available data found no increase in serious adverse events associated with Rotarix, RotaTeq, or Rotavac [32^▪▪^]. However, one consideration with all live-attenuated, oral rotavirus vaccines is the potential risk of intussusception, a rare but serious cause of bowel obstruction in which one portion of the intestine invaginates into another portion, in some cases necessitating surgery. The first rotavirus vaccine, RotaShield, was withdrawn from the US market in 1999 after it was found to be associated with risk of intussusception [33]. Further, the natural incidence of intussusception peaks during the same ages as rotavirus vaccination is given in many countries [34]. Although currently available rotavirus vaccines did not show an association with intussusception during clinical trials, the rarity of this outcome makes it difficult to evaluate without extremely large numbers of participants.

Postmarketing surveillance in high-income and middle-income countries has detected a temporally limited but significant increase in the risk of intussusception in the 1–7 days following administration of Rotarix or RotaTeq [35–40], on the order of 1 to 6 excess cases per 100 000 infants vaccinated. However, results from high-income and middle-income countries may not be fully generalizable to low-income and lower middle-income countries, which demonstrate important differences in access to healthcare as well as rotavirus vaccine effectiveness, as previously discussed. Further, the baseline incidence and epidemiology of intussusception may also vary by country. Lastly, it cannot be assumed that the risk profile will be the same across all rotavirus vaccines.

Recent data from an intussusception surveillance network in seven Rotarix-using African countries showed no significant increase in intussusception risk following rotavirus vaccination [41^▪^]. Infants were enrolled if they were less than 12 months of age and had an intussusception meeting the Brighton Collaboration level 1 diagnostic criteria. Information on clinical characteristics, demographics, and vaccination dates and status were collected, and the self-controlled-case series method was used for analysis. Overall, no excess risk of intussusception was detected following the first or second doses of rotavirus vaccine, in any of the risk windows studied (up to 21 days postvaccination). Although it is unknown why these results differ from previous studies in other settings, the authors propose several hypotheses. Rotavirus vaccines are known to be less efficacious in lower resource settings. If intussusception following rotavirus vaccination is related to the replication of the vaccine, and thus, efficacy, then this could explain differences in intussusception risk. In low-income countries, rotavirus vaccine is given concurrently with oral polio vaccine (OPV), which has been shown to inhibit immune response to rotavirus vaccine [42–44]. If vaccine-associated intussusception is associated with vaccine immunogenicity, this could again be a factor given that OPV is no longer used in high-income countries. In the majority of countries in this study, the immunization schedule calls for vaccination at 6 and 10 weeks of age -- earlier than many high-income countries, which often schedule the vaccine at 2 and 4 months of age. Given that the natural incidence of intussusception is extremely rare in very young infants, this schedule difference could play a role in the contrasting results. The authors acknowledge that there may be other differences in the study settings that could play a role through as-yet-unknown mechanisms -- for instance, infant microbiome, feeding and weaning practices, or maternal antibody levels.

Given the importance of this research question, the wide variability in intussusception epidemiology by geography, and the possibility that associations could vary by vaccine product, ongoing investigation is necessary. In this vein, surveillance is ongoing in two multicountry networks: postintroduction intussusception surveillance is currently underway in several African countries using RotaTeq [45], whereas baseline (preintroduction) and postintroduction intussusception surveillance is ongoing in several Asian countries, including in India (for the newly prequalified vaccines) [46,47]. Results from these evaluations will be helpful in further understanding the risk–benefit profile of rotavirus vaccines globally.

## NEWLY WHO-PREQUALIFIED ROTAVIRUS VACCINES

The availability of more affordable rotavirus vaccines will be an important part of ensuring continued coverage into the future. In 2018, two new rotavirus vaccines were prequalified by WHO: Rotavac and ROTASIIL. These vaccines will soon be available for use globally, but are currently only in routine use in India. This section will, thus, focus on the safety and efficacy data available from randomized controlled trials.

### Rotavac (Bharat Biotech)

Rotavac is a monovalent G9P[11] naturally attenuated, live oral rotavirus vaccine. A randomized, double-blind, placebo-controlled trial evaluated the safety and efficacy of this vaccine in more than 6500 infants enrolled from three sites in India [30]. Infants were randomized to receive either vaccine or placebo at a target schedule of 6–7, at least 10, and at least 14 weeks of age, and were followed up to 2 years of age for adverse events and gastroenteritis. Infants received routine immunizations as regularly scheduled (i.e. on the same day as the study vaccine). The estimated efficacy of Rotavac against severe rotavirus gastroenteritis requiring hospitalization or supervised rehydration was 56% (95% CI: 37–70%) in the first year of life [30] and 49% (95% CI: 17–68%) in the second year of life [48]. The occurrence of adverse events was not significantly higher in the vaccine group as compared with the placebo group; however, there was insufficient power to evaluate an association with intussusception [30,48]. In a noninterference trial (also randomized, double-blind, and placebo-controlled), no difference was seen in the immune response to pentavalent or OPV vaccines when comparing Rotavac recipients to placebo recipients [49].

### ROTASIIL (Serum Institute of India)

ROTASIIL, a pentavalent bovine-human reassortant live attenuated oral vaccine, has the unique feature of being heat-stable in its lyophilized form, retaining stability for up to 18 months at 40 °C [50]. This vaccine was evaluated in two randomized, double-blind, placebo-controlled trials: one in Niger [51] and one in India [29]. In each trial, infants were randomized to receive either vaccine or placebo at a target schedule of 6, 10, and 14 weeks of age, in coordination with any other vaccines recommended by the Expanded Program on Immunization (EPI) schedule. In Niger, the vaccine was transported and stored centrally at up to 25 °C until distribution to health centers, at which time it was stored at ambient temperature. In both trials, children were followed for serious adverse events and episodes of acute gastroenteritis; primary analyses were conducted after a target number of cases had been reached. In Niger, the vaccine efficacy against severe rotavirus gastroenteritis was 67% [95% CI: 50–78%; per-protocol analysis at the time of primary analysis (event-driven cut-off)], and no significant differences in adverse event rates were noted in the vaccine as compared with the placebo group, though the study was not powered to evaluate differences in the risk of intussusception [28,51]. In India, the vaccine efficacy against severe rotavirus gastroenteritis in the first year of life was 33% (95% CI: 12–49%; per-protocol analysis). The proportion of infants experiencing adverse events was similar in the vaccine and placebo populations; however, the study was not powered to detect significant differences in the risk of intussusception [29]. No evidence was found of interference with routinely administered EPI vaccines [52].

### Current usage of Rotavac and ROTASIIL in India

Both Rotavac and ROTASIIL are being introduced on a phased basis in India. As of 2019, Rotavac has been introduced into the Expanded Program on Immunization (EPI) schedule in 10 states: 4 in 2016, 5 in 2017, and 1 in 2018 [53]. Approximately 50 million doses have been procured by the Ministry of Health and Family Welfare so far [54]. In 2018, ROTASIIL was introduced into one state, and over one million doses have been distributed since then [55]. Postmarketing studies to evaluate the safety of the vaccines with respect to intussusception and the vaccine effectiveness are underway, and the expansion of routine use of both vaccines is anticipated with country-wide rollout projected by late 2019.

## NATIONALLY LICENSED VACCINES

### Rotavin-M1 (POLYVAC)

Nationally licensed vaccines can play an important role in broadening the affordability and availability of rotavirus vaccination, particularly in countries with the desire and ability to pursue local manufacture. For instance, in Vietnam, the government has made a concerted effort to foster the development and local manufacture of vaccines, to enable self-sufficiency [56]. Rotavirus-focused efforts led to the development of Rotavin-M1, a live, attenuated, frozen oral vaccine derived from a human rotavirus strain (G1P[8]) isolated from a child hospitalized for diarrhea in Nha Trang, Vietnam [56,57]. This vaccine was shown to be well tolerated and immunogenic (73% seroconversion) in a trial of Vietnamese infants [57]. Rotavin-M1 has been licensed on this immunogenicity data in Vietnam since 2012 and is available in the private market, with a two-dose schedule at 2 and 4 months of age. This vaccine is currently being introduced into the EPI schedule on a pilot basis in selected districts of two provinces: Nam Dinh and Thua Thien Hue. Vaccine effectiveness and impact evaluations are ongoing. A phase III immunogenicity trial of a liquid, nonfrozen presentation of the vaccine is being planned [58].

### Lanzhou lamb rotavirus vaccine (Lanzhou Institute of Biological Products)

The Lanzhou lamb rotavirus vaccine (LLR) is a live, attenuated oral vaccine based on a lamb rotavirus strain (G10P[15]) first isolated in 1985, and is licensed exclusively in China [59,60]. LLR has been available in China since 2000, and more than 60 million doses have been distributed [61]; however, the vaccine is not part of the country's national immunization program, and consequently, coverage is relatively low [62]. Further, coverage is highly variable by geography. LLR is recommended to be given once annually for children 2 months to 3 years of age. Although no efficacy data are available, as no placebo-controlled phase III trials were performed for this vaccine, several case–control studies have estimated vaccine effectiveness against rotavirus gastroenteritis. These studies, from different time periods and different geographies, generated vaccine effectiveness estimates ranging from 35 to 77% [59,63–65]. More recently, an ecological analysis suggested that the incidence of rotavirus gastroenteritis among young children was reduced in districts with higher as compared with lower rotavirus vaccine coverage [62]. Further research could be useful in evaluating the performance of and optimal schedule for this vaccine.

## ROTAVIRUS VACCINE PIPELINE

**FIGURE 2:**
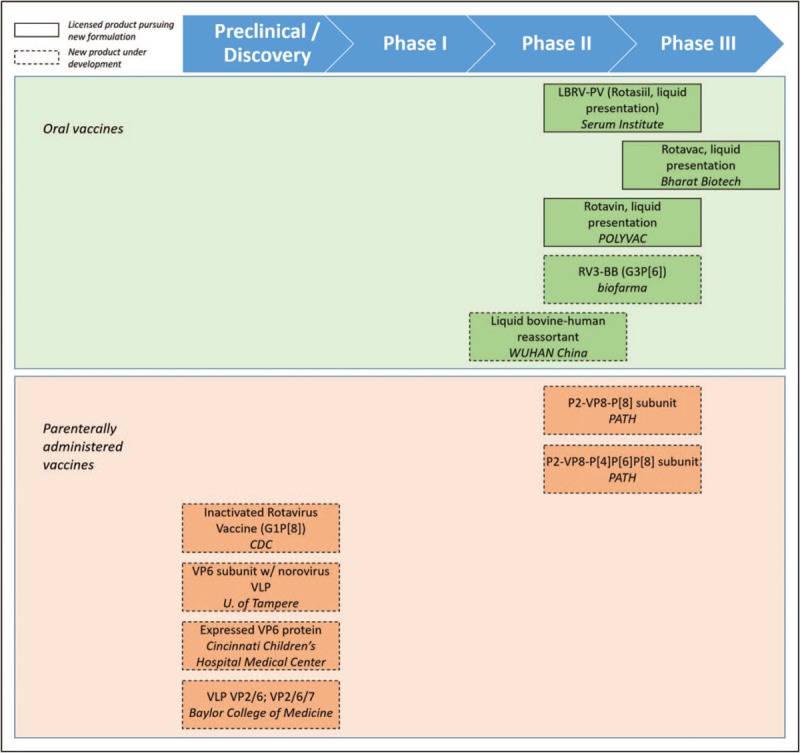
Rotavirus vaccine products in active development. Rotavirus vaccine products are shown by stage of development (horizontal position corresponding to labeled chevrons) and type (color). Oral vaccines are shown in green, whereas parenterally administered vaccines are shown in orange. Dashed lines indicate completely new products under development, whereas solid lines indicate licensed products pursuing new formulatons.

Several rotavirus vaccine candidates are in the pipeline [66], including further development of licensed products or strains, as well as new strategies to overcome some of the challenges associated with live, attenuated, oral infant vaccines (Fig. 2). This section will provide a brief overview of products actively under development.

### RV3-BB

The candidate that is furthest along in development is RV3-BB (PT BioFarma, Bandung, Indonesia), an oral vaccine based on a naturally attenuated neonatal strain of G3P[6] rotavirus and initially developed by the Murdoch Children's Research Institute [67^▪▪^]. RV3-BB, because of its ability to replicate in the newborn gut even in the presence of maternal antibodies, is intended to be given on a neonatal schedule with the goal of providing early protection against rotavirus [67^▪▪^]. The most recent data for this vaccine is from a Phase 2b, randomized, placebo-controlled trial that took place in Indonesia from 2013 to 2016. Full-term infants (*N* = 1649) were randomly assigned to one of three arms: placebo, neonatal schedule (0–5 days, 8–10, and 14–16 weeks of age), or infant schedule (8–10, 14–16, and 18–20 weeks of age); all infants received either placebo or vaccine, according to their trial arm, at each of the four time points. Infants were followed up to 18 months of age for severe rotavirus gastroenteritis. The per-protocol vaccine efficacy against severe rotavirus was 94% (56–99%) at 12 months of age for infants receiving vaccine on the neonatal schedule, and 77% (31–92%) at 12 months of age for infants receiving vaccine on the infant schedule. Immune responses were also noted in both groups, and the vaccine was well tolerated in both vaccine groups [67^▪▪^]. Further, no evidence was noted of interference by or with OPV in the neonatal schedule group [68]. This vaccine is being further evaluated in a dose ranging study in African neonates and infants [69]. Biofarma are currently driving clinical development, with the aim to introduce the vaccine into the Indonesian national immunization program by 2021 and eventually pursue a product for the global market.

### Nonreplicating rotavirus vaccines

Another strategy currently being investigated is the development of parenterally administered rotavirus vaccines. Such vaccines could have the potential to overcome some of the challenges associated with oral vaccines, such as interference by neutralizing antibodies present in breast milk, and other barriers associated with reduced efficacy [70^▪^]. Further, parentally administered vaccines could be combined with other infant immunizations. One such nonreplicating rotavirus vaccine (NRRV) under development is the subunit vaccine P2-VP8-P[8], most recently assessed in South African toddlers and infants [71]. During a dose-escalation phase, 90 toddlers and infants were randomized to receive vaccine or placebo; doses of 30 and 60 μg were tolerated and selected for further study in an expanded group of 114 infants, again randomized to receive vaccine or placebo. The vaccine was well tolerated, and vaccinated infants demonstrated strong IgG response (>98% seroconversion) compared with placebo (9% seroconversion) [71]. More recently, a trivalent subunit vaccine (VAC 041, P2-VP8-P[4]P[6]P[8]) was studied using a similar design [72]. Strong IgG responses were demonstrated, and neutralizing antibody response to several strains of rotavirus was also noted.

There is also a VP6 subunit vaccine under development that incorporates norovirus virus-like particles (VLPs) to form a combination vaccine [73–76]. This candidate has demonstrated good immunoresponse in mice models [75,76]. An inactivated rotavirus vaccine (IRV) has also been developed by CDC for parenteral administration [77]. This candidate, based on a G1P[8] strain, has been trialed in mice, rats, rabbits, and pig models and demonstrated heterotypic neutralizing antibody response [78,79]. Systemic and mucosal immunity were shown in mice after administration by injection and by microneedle patch [77]. Further, this candidate was efficacious against oral rotavirus challenge in piglets [80]. A combined inactivated polio vaccine (IPV)–IRV product is also under development with this same strain, and has been tested in mice, with no evidence of interference of the immune response to either component [81]. Other early-stage candidates include an inactivated G1P[8] strain isolated from a hospitalized Chinese infant [82] and a truncated VP4 based on the LLR strain [83].

## CONCLUSION

Now, with four WHO-prequalified oral rotavirus vaccines available, and several more products nationally licensed or in development, we are at an unprecedented time of choice in the history of rotavirus vaccines. However, rotavirus disease remains a large burden worldwide, and numerous countries have yet to introduce rotavirus vaccines into their national schedules. Rotavirus vaccines have a proven track record of impact, balanced with a favorable risk–benefit profile. With new products in the pipeline, and several countries poised to introduce vaccines formerly available only in a single market, we look forward to the continuation of an exciting era in the use of rotavirus vaccines.

## Acknowledgements

None.

Disclaimer: The findings and conclusions of this report are those of the authors and do not necessarily represent the official position of the Centers for Disease Control and Prevention (CDC). Names of specific vendors, manufacturers, or products are included for public health and informational purposes; inclusion does not imply endorsement of the vendors, manufacturers, or products by the Centers for Disease Control and Prevention or the US Department of Health and Human Services.

### Financial support and sponsorship

None.

### Conflicts of interest

There are no conflicts of interest.

## REFERENCES AND RECOMMENDED READING

Papers of particular interest, published within the annual period of review, have been highlighted as:

▪ of special interest
▪▪ of outstanding interest
